# Shape based indexing for faster search of RNA family databases

**DOI:** 10.1186/1471-2105-9-131

**Published:** 2008-02-29

**Authors:** Stefan Janssen, Jens Reeder, Robert Giegerich

**Affiliations:** 1 Faculty of Technology, Bielefeld University, 33615 Bielefeld, Germany

## Abstract

**Background:**

Most non-coding RNA families exert their function by means of a conserved, common secondary structure. The Rfam data base contains more than five hundred structurally annotated RNA families. Unfortunately, searching for new family members using covariance models (CMs) is very time consuming. Filtering approaches that use the sequence conservation to reduce the number of CM searches, are fast, but it is unknown to which sacrifice.

**Results:**

We present a new filtering approach, which exploits the family specific secondary structure and significantly reduces the number of CM searches. The filter eliminates approximately 85% of the queries and discards only 2.6% true positives when evaluating Rfam against itself. First results also capture previously undetected non-coding RNAs in a recent human *RNAz *screen.

**Conclusion:**

The **RNA s**hape **i**ndex **f**ilter (*RNAsifter*) is based on the following rationale: An RNA family is characterised by structure, much more succinctly than by sequence content. Structures of individual family members, which naturally have different length and sequence composition, may exhibit structural variation in detail, but overall, they have a common shape in a more abstract sense. Given a fixed release of the Rfam data base, we can compute these abstract shapes for all families. This is called a shape index. If a query sequence belongs to a certain family, it must be able to fold into the family shape with reasonable free energy. Therefore, rather than matching the query against all families in the data base, we can first (and quickly) compute its feasible shape(s), and use the shape index to access only those families where a good match is possible due to a common shape with the query.

## Background

### ncRNA

Computational screens [1-3] predict thousands of potentially conserved secondary structures in the human genome. Similar screens in Yeast and Nematodes [4] also produced thousands of potential non-coding RNAs (ncRNAs). Some were already identified by sequence comparison as members of the known RNA families, but the meaning of the majority remains unclear. With functional RNA, structure is often more important than primary sequence content. Thus, a BLAST screen against RNA sequence databases is not enough – the structure needs to be taken into account when searching for known relatives of a query RNA.

#### Rfam and its usage

The Rfam database [5] is a constantly growing data source for ncRNAs. The current release (Rfam 8.0, February 2007. During the submission process a new version of Rfam (8.1, 607 families) appeared.) contains 273 989 annotated sequences grouped in 574 families. An Rfam family contains two secondary structure annotated, multiple alignments, a so called *seed *alignment and a larger *full *alignment. A consensus structure is also provided (annotated as *SS_cons*), with no guarantee that family members actually fold into this consensus. The seed alignment usually is hand-curated and contains only validated sequences from the literature and other databases. From the seed alignment, a probabilistic model is learned, which is then used to annotate new members of this family. All members with a good CM (covariance model) score build the full alignment.

Researchers today use the information stored in Rfam routinely to annotate newly sequenced bacterial genomes, and with caution also for more complex eukaryotic genomes. The major obstacle researchers encounter hereby is the high computational complexity, which makes the annotation process very time consuming or even impossible. Filtering techniques are therefore necessary to speed up the analysis. In the next sections we will shortly review the probabilistic models used for searching and two sequence based filtering techniques.

#### Searching with covariance models

*Covariance models *(CMs) [6] are probabilistic models, incorporating family specific structural information, much like profile hidden Markov models (HMM) in (linear) sequence analysis do. The main advantage of a CM over a HMM is, that it can model the long range interactions we see with RNA base pairing.

CMs basically are profile *stochastic context free grammars *(SCFG). Each base pair and each unpaired residue is represented by one *state*. States are arranged in a tree-like structure that mirrors the tree-like consensus structure of an RNA family. Additional states model insertions and deletions of bases, differing from the consensus. Transitions from one state to another in a CM are modeled by production rules, each having certain transition and emission probabilities, learned from a multiple structural alignment. Given a CM and a query sequence, the algorithmic problem is to find the path through the CM that emits the sequence with the highest probability. This can be done efficiently using dynamic programming with a CYK-like parsing algorithm in *O*(*LN*^3^), where *L *is the target sequence length and *N *the window size. The programs to build and search with CMs are bundled in the software package *Infernal*.

Despite some recent improvements [7], many Rfam families still require more than an hour CPU time per Mb. Consequently, a large eukaryotic genome cannot be annotated in total within reasonable time. The search procedure has to be restricted to the most promising regions. This is where the need for efficient filters arises.

### Filtering

#### Basic concepts of filtering

Usually, filters strive to rule out as much of the input data as possible, without discarding too many positive cases. Of course, these are two competing goals and in practice a trade-off has to be made. We define the ratio of the input data that passes the filter and the total input data as the *filtration ratio*. A good filter has a low filtration ratio, a useless one a ratio of 1. The *sensitivity *is defined as the ratio of positives passing the filter and all positives. A special case are *rigorous *filters, which have a perfect sensitivity. They never filter out a true positive.

**Figure 1:**
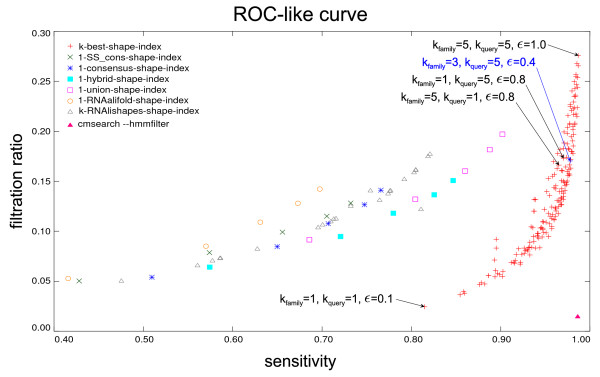
**Evaluation for the different shape indices**. The app. 10,000 sequences of the testing set are searched against Rfam with all 5 mentioned types of shape indices, that are namely: 1-SS_cons-, 1-consensus-, 1-hybrid-, 1-union-, and k-best-shape-index. Additionally the figure contains results for a 1-RNAalifold-shape-index (RNAalifold is used to construct a consensus structure for each family that is later transformed to a shape), a k-RNAlishapes-shape-index (the same as RNAalifold but with suboptimal structures of different shape), and the results from a complete run with the HMM filter (pink triangle in the lower right corner). 175 different parameter sets, see subsection Testing in the Evaluation, are used for the k-best-shape-index. The blue coloured data point (*k*_*family *_= 3, *k*_*query *_= 5, *ε *= 0.4) displays our recommended parameter settings as a practical trade-off between filtration ratio and sensitivity. *k*_*family *_for the k-RNAlishapes-shape-index is handled as before, but the energy-index is switched off by a gigantic value for *ε*. The other five shape-indices use only one shape per family or per family member, so their *k*_*family *_is always set to 1. Their energy-index is switched off, too.

With most non-rigorous filters, we can easily change sensitivity versus filtration ratio, i.e. accuracy versus speed. Plotting these two values, yields a ROC-like curve (ROC = receiver operating characteristic, a graphical plot of the sensitivity vs. (1 – specificity), see Figure 1), which allows for an easy comparison of different filters over a wide range of parameters.

#### BLAST-filter

The curators of Rfam provide a simple and fast BLAST based filtering heuristic [8]: All BLAST hits with a P-value *<*10 to any member of the seed alignments are extracted and a family specific window size is added to both ends of the hit. Only the much smaller subsequences are then analyzed with an expensive CM search. Despite its simplicity, the filter has been used to add sequences to Rfam. For each new Rfam release, a portion of the EMBL nucleotide database is scanned this way. The resulting CM hits, together with the seed members, then constitute the full alignment.

The more diverse in sequence the seed alignment is, the more likely distantly related family members will be recognized. Also, with each new family member the filter becomes more sensitive, yet less specific. However by construction, an RNA perfectly in agreement with the consensus structure but very dissimilar in sequence will be discarded. At the moment, it is unclear how many homologues are hidden in the databases, overlooked by the BLAST filter.

#### HMM-filter

Recently, another filtering approach was introduced by Weinberg and Ruzzo. They suggest profile HMMs, automatically built from the CM, as a prefilter to the CM search. In [9], the authors demonstrate how to convert a CM into a rigorous HMM filter. First, the CM has to be linearized. For a base pair state *s *in the CM, emitting columns *i *and *j *in the multiple alignment, two states in the HMM are introduced, namely at position *i *and *j*. Now, assume the CM emits only C-G and G-C base pairs in state *s*, then the HMM would emit a C or G at position *i *and another C or G at position *j*. Of course the base pair condition cannot be modeled this way, but nevertheless the sequence information is still available. Second, the scores of the HMM are cleverly chosen, such that the HMM Viterbi score is an upper bound for the CM score. Thus any subsequence scoring below a certain threshold can be safely discarded. For many Rfam families the filtration ratio is *<*0.01, thus making the HMM scan itself the run time determining step. The HMM searches scan the database approximately 200 times faster than the original CM searches.

However, for some families the rigorousness requirement prevents a significant speed up of the resulting HMM over the CM. Also the filtering efficiency may suffer from the attempt to capture even the most excentric family member. A way out of this dilemma are heuristic filters (called Maximum-Likelihood (ML) heuristics in [10]), which sacrifice rigorousness for speed. The ROC-like curves in [10] and the pink data point in the lower right of Figure 1 give an impression of this fact. The current *Infernal *release 0.81 provides a variant of the ML-heuristic.

#### A new approach: shape based filtering

Our idea of **RNA s**hape **i**ndex **f**iltering (*RNAsifter*) is based on the following rationale: An RNA family is characterised by structure, much more succinctly than by sequence content. Structures of individual family members, which naturally have different length and sequence composition, may exhibit structural variation in detail, but overall, they have a common shape in a more abstract sense. Given a fixed release of the database, we can compute these abstract shapes for all families. This is called a shape index.

If a query sequence belongs to a certain family, it must be able to fold into the family shape with reasonable free energy. Therefore, rather than matching the query against all families in the data base, we can first (and quickly) compute its feasible shape(s), and use the shape index to access only the families where a match is possible due to a common shape with the query.

In an ideal world, this results in a rigorous filtering algorithm (rather than a heuristics): It reduces the number of searches (and therefore computation time), while no potential match is missed. In reality, the outcome depends on many details – the structural homogeneity of families, the type of shapes we compute, and so forth. Moreover, the general idea gives ample room for alternative implementations. We will explore some of these in the sequel, and end up with a quite effective (although not perfect) parameter set that finds 97.4% of all hits performing only 15.0% of the queries.

### A review of abstract shapes of RNA

Abstract shapes of RNA were introduced in [11]. We give a short review, avoiding a fully formal treatment. When we speak about functional RNA classes, we do not refer to concrete structures – we employ abstraction. A tRNA has a cloverleaf structure, a microRNA precursor is a lengthy hairpin, *oxyS *RNA has three adjacent hairpins. Obviously, the most important structural characteristic is the specific arrangement of RNA helices, governed by the two principles of *adjacency *and *embedding*. The cloverleaf, for example, is a helix which embeds three helices adjacent to each other. Sometimes, we want to be less abstract. The iron responsive element, for example, is a small hairpin with a bulged-out cytosin that is essential. The technique of abstract shape analysis [11] formalises the concept of shapes and teaches RNA folding programs to compute with these shapes. This is done in a mathematically precise sense, with no heuristics involved. The program *RNAshapes *computes the *k *≥ 1 near-optimal structures which have *different *shapes, thus giving a concise overview over a molecule's structural inclinations. These structures are called **sh**ape **rep**resentatives, *shrep *for short, as each is an optimal structure with respect to its shape.

Probabilistic shape analysis [12] computes Boltzmann statistics shape-wise, giving us the accumulated probability of all ways in which the given sequence can fold into (say) a cloverleaf shape. Being a cloverleaf shape with (say) 80% probability is much more handsome information than traditional MFE folding, as this is independent of sequence length and composition, and hence comparable between different sequences. Formally, a shape abstraction is a mapping from concrete RNA structures to abstract shapes. Concrete structures are modeled as trees, as frequently done in the RNA bioinformatics. This is natural, as trees incorporate the two principles of adjacency (among sibling nodes) and embedding (from a parent node to its children). Abstract shapes, then, are also trees, but containing less detail. Any mapping from structures to shapes that is a *tree homomorphism*, i.e. preserves adjacency and embedding, can be used as shape abstraction. It is our decision which structural feature is to be retained and what is to be abstracted from.

In [11], five abstraction functions *π *∈ {*π*_1_, ... *π*_5_} (that produce shapes of levels 1 through 5) were introduced. They all abstract from the length of helices and unpaired regions, but are more or less forgetful about the presence of structural features like bulges and internal loops.

These five abstractions are the ones also used within the present approach. Here we skip their formal definitions, and rather explain them by example. In doing so, we use a string representation of shapes akin to the dot-bracket strings that commonly encode concrete structures. We use square brackets to denote helices (or helix parts) and underscores for unpaired regions. Their precise meaning, however, depends on the shape level used.

**Figure 2:**
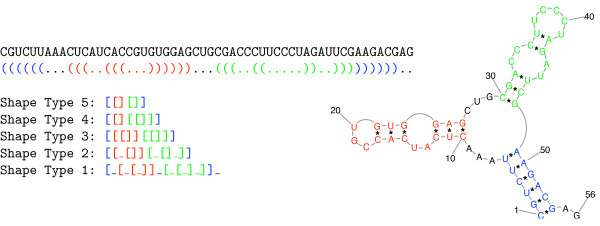
An example secondary structure and its five shape representations.

Figure 2 shows an example structure and its equivalent notation as dot-bracket string. Shape level 5 abstracts from all helix interruptions (bulge and internal loops) and ignores single stranded regions, such that the level-5-shape reduces the structure to a single helical region, bifurcating into two hairpins. With shape level 4, we account also for helix interuptions by internal loops (but not by bulges), and alternatively, level 3 records all helix interruptions, resulting in shape [[[]] [[]]]. Level 2 extends level 3, by also differentiating in between left and right bulges and finally in level 1 all continuous single stranded or helical regions are recorded explicitly.

Note that the level-5-shape [] comprises all structures with level-3-shape [], [[]], [[[]]], and so on – this is because on level 5, helix interruptions are not accounted for at all, while on level 3, each interruption of a helix by a bulge or internal loop implies an extra helix part recorded by an extra pair of square brackets.

Shape levels 1, ..., 5 are designed to form a strict hierarchy: If *π*_*i*_(*x*) = *π*_*i*_(*y*), then also *π*_*j*_(*x*) = *π*_*j*_(*y*) for *j *> *i*. (In fact, we found an error in this respect in the original implementation of *π*_2 _and *π*_4_, and corrected it in the course of this study. The hierarchy property is not strictly necessary for our filtering purpose here, but mathematically pleasing and useful in other applications of abstract shape analysis.)

## Methods

Throughout this section, we use Rfam as "the" RNA family database. Note, however, that our filtering technique applies to any database that groups RNA sequences into structurally related families.

### Shape-based indexing framework

Shape-based indexing works as follows:

1. For each family *f *∈ Rfam, we compute a *family shape spectrum fss*(*f*).

2. {*fss*(*f*) | *f *∈ Rfam} is converted into an index data structure *I*_Rfam _such as a hash table or a suffix tree, with search access time independent of the size of *I*_Rfam _and hence of Rfam size.

3. For a given query sequence *x*, we compute a *query shape spectrum qss*(*x*).

4. We access the index *I*_Rfam _to determine the *match set M*(*x*) = {*f *| *qss*(*x*) ⋂ *fss*(*f*) ≠ ∅}.

5. If *M*(*x*) = = ∅, the query ends with a negative result, without access to Rfam. Otherwise, we execute *cmsearch*_*f*_(*x*) for each *f *∈ *M*(*x*), which determines the outcome of the query.

Index construction (steps 1 and 2) must only be performed once for each release of the data base. The matching against the index (step 4) requires an *exact *match of a shape in *qss*(*x*) to a shape in some *fss*(*f*). This makes access to the index so fast that its execution time is negligible compared to a call to *cmsearch*. The shape spectra *qss*(*x*) and *fss*(*f*) can be computed in many different ways, and can be combined (almost) arbitrarily in indexing. We describe several variants and finally report which combination of ideas has worked best after extensive evaluations.

Alternative shapes of a sequence, as computed by *RNAshapes*, are ranked according to the free energy of their respective shreps. We denote by *RNAshapes*(*k*, *π*, *x*) the computation of the *k *top-ranked shapes of *x *under the shape abstraction function *π*, where *π *∈ {*π*_1_, ... *π*_5_}. *k *= 0 means we compute all shapes for *x*. By *π*(*s*), we denote the shape of structure *s*. *RNAfold*_-*C*_(*a, x*) [13] denotes the minimum free energy structure of *x *under the constraint that the base pairs indicated in the annotation string *a *must be formed.

### Shape index construction

#### 1-SS cons-shape-index: *fss*(*f*) = {*π*(*SS*_*cons*)}

The simplest way to get a shape abstraction for each Rfam family is by translating the already given secondary structure consensus – that is the SS_cons row in the family alignment – with *RNAshapes *to one single shape. *RNAshapes *is not able to deal with pseudoknots, so these sparsely occurring structures must be resolved before by unpairing crossed over basepairs.

#### 1-consensus-shape-index: *fss*(*f*) = *rankmin*{⋂_*x*∈*f *_RNAshapes(0, *π*, *x*)}

Evaluations revealed that the given SS_cons in Rfam often is not a really consensus structure in terms of a commonly shared shape. There are even a few families where not a single member folds in the shape *π*(SS_cons)! This can be caused by many reasons, e.g. inaccuracies in the thermodynamic model, too inhomogeneous families, or misbuilt families. Therefore, we construct consensus-shapes directly from the family *f*. First we calculate all possible shapes for each family member. Then we scan for shapes common to all family members. If there are more than one common shapes, they are ranked by the sum of their individual ranks. The top-ranked common shape then represents the family in the index. This resembles the *RNAcast *approach to consensus structure prediction [14]. Using a trusted sequence alignment, this could also be done with *RNAalifold *[15] or *RNAlishapes *[16].

#### 1-hybrid-shape-index

When a family is large, sequences are long, heterogeneous in structure, and shape abstraction level is low, the number of shapes to be computed to find a common shape may be impractical. In such a case, the 1-consensus-shape-index construction resorts to 1-SS_cons-shape indexing.

#### union-shape-index: *fss*(*f*) = {*π*(RNAfold__*C*_(*SS*_*cons*, *x*)) | *x *∈ *f*}

The first three approaches all use a single shape to represent a family. But often, a family is too diverged to be characterized well by a single shape. Instead, it can be described with one shape per sequence. We could simple use the shape of the MFE folding. However, to make use of the information in the family model, as captured by SS_cons, we use RNAfold for a constrained folding of each sequence, and compute the shapes from these folds. This implies that all shapes agree on the helices required by SS_cons, but may have additional helices in different places. We still expect |*fss*(*f*)| < |*f*|.

#### k-best-shape-index: *fss*(*f*) = ⋃_*x*∈*f *_RNAshapes(*k*, *π*, *x*)

**Figure 3:**
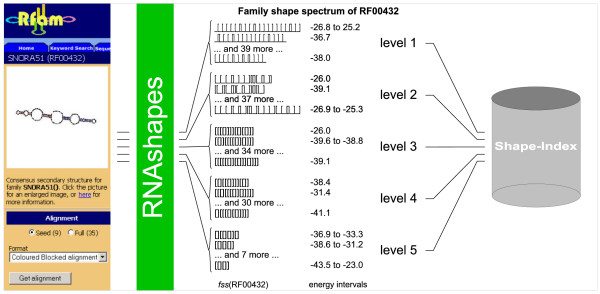
**Workflow of a shape index construction**. The example demonstrates the process of index construction for the family RF00432. For each of the five shape abstraction levels, an independent shape index has to be computed. For this purpose, every single sequence of each Rfam family (currently 574 families in Rfam 8.0) has to be abstracted with *k*_*family *_shapes (*k*_*family *_= 2 for the example). This is done by *RNAshapes*. After the shapes were computed, they are stored in the hash based shape index, where they later serve for the query look-ups. The index is a hierarchically arranged, 3-dimensional search structure – in descending search order of shape-abstraction-level, shape-string and shrep-energy. The example also displays a very characteristic phenomenon: the weaker an abstraction is, the greater is the variance of shapes and the smaller are the family-lists that are associated with these shapes. The last fact is not shown directly, but can be recognized by the increasing energy-interval-size.

Respecting the fact that a single shape may be too strict to describe a whole family, we finally use the k top-ranked shapes for each individual sequence in the family, this time ignoring SS_cons. In a homogeneous family, the sequences will mostly agree on these shapes. But otherwise, this results in a manifold growth of the shape index – which is not a problem, as index access is independent of index size. In the end, this index turned out as the most effective. Figure 3 visualizes the shape index construction for just one of the 574 Rfam families, employing multilevel shape abstraction as explained below.

in the hash based shape index, where they later serve for the query look-ups. The index is a hierarchically arranged, 3-dimensional search structure – in descending search order of shape-abstraction-level, shape-string and shrep-energy. The example also displays a very characteristic phenomenon: the weaker an abstraction is, the greater is the variance of shapes and the smaller are the family-lists that are associated with these shapes. The last fact is not shown directly, but can be recognized by the increasing energy-interval-size.

### Query shape spectrum construction

The five methods for index shape spectrum construction differ in the way they use family information, or effectively treat each family individually. For the query shape spectrum, we have only one sequence, and discuss only two variants.

#### 1-shape-spectrum: *qss*(*x*) = RNAshapes(1, *π*, *x*)

This naive spectrum represents the query simply by its top-ranked shape, which by definition is the shape of its minimum free energy folding.

#### k-shape-spectrum: *qss*(*x*) = RNAshapes(*k*, *π*, *x*)

We tend not to trust solely in the minimum free energy folding of a sequence as its "true" structure. Accordingly, we should not use its shape alone. Choosing *k > *1 shapes to represent the query results in a trade off between more shapes for a higher chance to find the right family, but potentially more fruitless calls to *cmsearch*.

### Using multi-level abstraction

All the previous constructions of family and query shape spectra can be used in combination, under the restriction that both were made using the same shape abstraction function. But which abstraction level should be used? How does the level of abstraction influence index-based search? On the lower abstraction levels (levels 1, 2), shapes are most specific. Relatively few families are associated with each shape. A shape match on this level often leads directly to the "right" family. However, if there is no match on the low abstraction level, the sequence may be a diverged family member, and a match on a higher abstraction level is still possible.

To take advantage of both, short runtime at low abstraction level and better chances to find diverged families with a strong abstraction, we construct *fss*(*f*) and *qss*(*x*) for level 1 through level 5, and the search iterates ascendingly through the five available abstraction levels. The hope is, that on average, a match can be found in one of the lower levels. Ascending through shape abstraction levels incurs practically no overhead – an unsuccessful CM search on (say) level 2 means that we need not re-search this family because of its hits to the index on a higher level.

### Using folding energies

**Figure 4:**
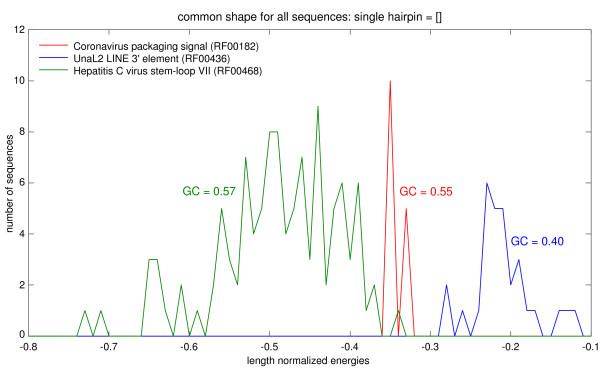
**Distribution of shrep energies, normalized to sequence length for three selected Rfam families**. The MFE structure for all sequences from the three chosen families is the single hairpin shape. While their abstract shape is the same, they differ in folding energy. To eliminate the influence of sequence length on the folding energy, it is divided by sequence length. The x-axis represents this normalized energy value and the y-axis shows the amount of sequences that fold with this normalized energy value. Several distinct peaks can be seen, showing that the shrep energy does not only depend on sequence length. Therefore, it provides an additional attribute of a family that can be used in filtration.

Members of sequence families often share a typical range of folding energies. A query that folds in a common shape with some family members, but with substantially different energy, is unlikely to be a family member. Together with the shapes, *RNAshapes *also delivers the energies of the corresponding shreps. Hence, the shapes in the index can be recorded together with their shrep energies, and the matches in *M*(*x*) are restricted to those with a similar energy. Figure 4 shows a very clear example. While all three families share the same shape – a simple hairpin – their energies form quite distinct energy ranges, independent of sequence length and GC-content. To this end, we reduce the match set *M*(*x*) to those families *f *that share a shape of their *fss*(*x*) and the *qss*(*x*) with the shreps free energy tolerance between both shapes less than *ε *percent.

this normalized energy value. Several distinct peaks can be seen, showing that the shrep energy does not only depend on sequence length. Therefore, it provides an additional attribute of a family that can be used in filtration.

### Omitting "difficult" families

Our evaluation shows that there are a few families which can hardly be found. Often (10 of 25 cases in Additional file 1), these are families with pseudoknots. Shape abstraction can, in principle, be extended to pseudoknots and implemented in pseudoknot folding programs such as *pknotsRG *[17,18]. However, this has not been done yet. For the moment, it is not surprising that these families perform badly. In a large scale project, some more runtime can be saved by ignoring these families. Additional file 1 lists the most difficult families, sorted by their impact on the filter sensitivity.

### Algorithm

The idea of shape-based indexing, as we have seen in the previous section, opens up a four-dimensional search space: We may vary the construction of family shape spectra, query shape spectra, shape abstraction level, and consider different tolerances in the use of energies. There are many trade-offs. For example, when the family is represented by several shapes per family member, chances increase that describing the query with a single shape is sufficient.

We have explored numerous points in this methodical space, and for the routine application considered here – matching a large number of predicted ncRNAs as queries against Rfam – the choice of methods described in this section has worked best.

#### Method of choice

The method of choice for our program *RNAsifter *is a combination of the k-best-shape-index, together with the multilevel abstraction, use of the folding energies and a k-shape-spectrum of the query:

$$\begin{array}{c} qss(x)=RNAshapes(k_{query},\pi ,x)\forall \pi \in \{\pi_{1},...,\pi_{5}\}\\ fss(f)=\underset{x\in f}{\cup }RNAshapes(k_{family},\pi ,x)\pi \in \{\pi_{1},...,\pi_{5}\}\\ M(x)=\{f|qss(x)\cap fss(f)\neq 0,\exists q\in qss(x),d\in fss(f)|E(q)-E(d)|<\varepsilon ,\} \end{array}$$

where *E *(*q*) denotes the shrep energy of shape *q*.

**Figure 5:**
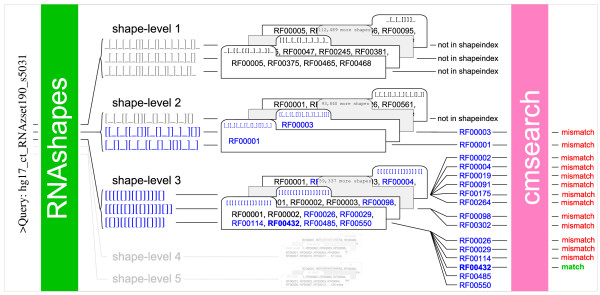
**A typical work flow of RNAsifter**. The filtering process is iterative. It starts with shape abstraction level 1 and ascends up to level 5, if it is not terminated earlier by a successful *cmsearch *of some family. At first all *k*_*query *_shapes in the current level are computed for the query by *RNAshapes*. In the example, *k*_*query *_is set to 3. Subsequently, each shape is used for a look-up in the shape index. All three level 1 shapes are not contained in the corresponding level-1-shape index so the system goes to the next higher level. A lookup for the second and third level 2 query shape (blue coloured) results in the two candidate families RF00003 and RF00001. The exact but expensive *cmsearch *is now applied to the candidates but it turns out that they are not the right families. So the process continues with level 3. This time all three shapes match with the level 3 shape index and suggest six, two and six candidate families. Two of the six candidates from the upper shape have been checked already, so they can be removed from the candidate list to save runtime. Again, a *cmsearch *is performed for each of the remaining 13 candidates. After the candidate RF00432 (bold blue coloured) is identified as a true hit, the filter aborts the remaining operations and reports the identified hit to the user. The use of the shrep energies and the energy-indices is not shown here, but one can imagine it as a second lookup placed between the first query-shape look up and the *cmsearch *in order to thin out the candidate list.

This combination of methods is implemented by *RNAsifter *[19], available as source code or for online submission. Figure 5 shows the iterative workflow through the 5 abstraction levels. 11 parameters remain to adjust to find an acceptable trade-off between sensitivity and filtration ratio, namely the number of shapes for the query $k_{query}^{\pi }$ for all five shape-levels, five parameters for the numbers of shapes for each family to build the shape index $k_{family}^{\pi }$, and the allowed energy tolerance between query-shape and the family-shape *ε*. It is optional to omit "difficult" families due to the decision of the user. This is realized by removing bad families from the set *M *(*x*) before applying *cmsearch*_*f *_(*x*).

the corresponding level-1-shape index so the system goes to the next higher level. A lookup for the second and third level 2 query shape (blue coloured) results in the two candidate families RF00003 and RF00001. The exact but expensive *cmsearch *is now applied to the candidates but it turns out that they are not the right families. So the process continues with level 3. This time all three shapes match with the level 3 shape index and suggest six, two and six candidate families. Two of the six candidates from the upper shape have been checked already, so they can be removed from the candidate list to save runtime. Again, a *cmsearch *is performed for each of the remaining 13 candidates. After the candidate RF00432 (bold blue coloured) is identified as a true hit, the filter aborts the remaining operations and reports the identified hit to the user. The use of the shrep energies and the energy-indices is not shown here, but one can imagine it as a second lookup placed between the first query-shape look up and the *cmsearch *in order to thin out the candidate list.

## Results and Discussion

Leaving out the BLAST filtering, the existing search process for a query sequence uses all available covariance models from the Rfam database and compares them to the query via the *cmsearch *program. The Rfam is an increasing set of *R *covariance models, for Rfam 8.0 *R *is 574. So the runtime for one query would be *O*(R * n^4^), in the worst case. Our approach reduces *R *to *r*_*Sifter*_, the number of models that have to be considered for a *cmsearch *comparison.

### Construction of a testing set

Performance of a filtering system is governed by sensitivity and filtration ratio. To measure sensitivity, one needs a set of queries with known family membership. We build this set by dividing all Rfam full alignments into two disjoint sets. 60% sequences from each family are used for constructing the shape index. From the remaining 40% of the family-sequences we choose at most four sequences for inclusion in the test set. This procedure yields 2030 test sequences in total, because some smaller families lack sufficient members. This set is named "family".

The second value for filtering performance is filtration ratio. In a whole genome screen, in most cases, a query will not result in a match to Rfam. The filtering suggests several possible families, which later have to be checked by *cmsearch*. Queries that result in a true match may lead to success after inspecting only a few suggested families, but queries with no family membership could only be rejected after checking each family that was recommended by the filter. This is why for evaluating efficiency, non-matching samples in the testing set are important. They constitute the worst case in an application scenario.

The most obvious way of constructing non-matching sequences is to generate them by random. Each nucleotide occurs with a relative frequency of 1/4. The length of the artificial sequences is uniformly distributed in the range from the shortest to the longest sequence in the Rfam database. 2000 random sequences build the "random_uniform" testing set.

In order to achieve a biological more realistic scenario, 11 protein-coding genes, considered unlikely to form any ncRNA, were selected from NCBI. Subsequently these genes were verified to truly not contain any ncRNA structures via the Rfam sequence search. These genes serve as source for further 2000 sequences, randomly cut out of the original genes. Again, lengths are uniformly distributed relative to Rfam. This set is called "genes_uniform". The chosen genes have the following accession-numbers: gi|110225369, gi|85815826, gi|109148525, gi|45219732, gi|79476965, gi|20804396, gi|40515, gi|42568004, gi|23297153, gi|3005973 and gi|46559395.

The energy-index depends on the folding energies and the folding energies are related to the sequence lengths. The previous sampling sets, namely "random_uniform" and "genes_uniform", are based on uniformly distributed sequence lengths in the Rfam database. But this is not true for the database. Rfams longest sequence has approximately 850 bases, but the overwhelming majority has around 50 to 150 nucleotides. The two further test sets "genes_nonuniform" and "random_nonuniform" reflect this imbalance, because their sequence lengths are distributed equally to Rfam. Each set is a composition of 2000 sequences. All test sets are provided on the *RNAsifter *web site, the whole evaluation was done with the union of all test sets (10030 sequences).

### Testing

*RNAsifter *offers 11 parameters for choosing an acceptable trade-off between sensitivity and filtration ratio:

• Five parameters for the numbers of shapes for the query $k_{query}^{\pi }$. One for each shape abstraction function *π*_1_, ⋯, *π*_5_.

• Five parameters for the numbers of shapes for shape index construction $k_{family}^{\pi }$. One for each shape abstraction function *π*_1_, ⋯, *π*_5_.

• Percentage of tolerance between query- and shape index energy *ε*.

We decided to freeze the number of shapes in each level to the same value and to sample with the following energy tolerances 1.0, 0.8, 0.6, 0.4, 0.3, 0.2, 0.1. So *k*_*query *_is the number of shapes for a query in all five shape-levels. *k*_*family *_is the same for shape index construction. This results in 5·5·7 = 175 different parameter settings. Each setting is used for an *RNAsifter *invocation with the app. 10.000 test sequences. The sensitivity (*sen*) is then calculated as the ratio between true positive *RNAsifter *outcomes and the overall number of positive test sequences, that is 2030: $sen=\frac{TP}{2030}$.

Filtration ratio (*eff*) is the ratio between all candidate families suggested by the *RNAsifter *and the number of all sequences multiplied with the number of families in Rfam: $eff=\frac{\#candidates}{\#sequences\times \#families}$. RNAalifold but with suboptimal structures of different shape), and the results from a complete run with the HMM filter (pink triangle in the lower right corner). 175 different parameter sets, see subsection Testing in the Evaluation, are used for the k-best-shape-index. The blue coloured data point (*k*_*family *_= 3, *k*_*query *_= 5, *ε *= 0.4) displays our recommended parameter settings as a practical trade-off between filtration ratio and sensitivity.

*k*_*family *_for the k-RNAlishapes-shape-index is handled as before, but the energy-index is switched off by a gigantic value for *ε*. The other five shape-indices use only one shape per family or per family member, so their *k*_*family *_is always set to 1. Their energy-index is switched off, too.

The results for all 175 parameter settings are depicted in Figure 1. The relatively wide parameter space allows to adjust *RNAsifter *for different applications. For a very accurate search the given parameters could be set to very high values, like our analysed maximum *k*_*family *_= 5, *k*_*query *_= 5, *ε *= 1.0 – this is the right uppermost data point in Figure 1. This unbalanced setup results in a good sensitivity of 98.67%, but it must be bought by a poor filtration ratio of only 27.58%. Vice versa, a fast runtime comes with a lower sensitivity, e.g. the data point *k*_*family *_= 1, *k*_*query *_= 5, *ε *= 0.1 indicates a good filtration ratio of 5.99% and a low sensitivity of 90.25%. Our recommendation (blue data point in Figure 1) for a good trade-off between filtration ratio (15.03%) and sensitivity (97.44%) is the parameter setting *k*_*family *_= 3, *k*_*query *_= 5 and *ε *= 0.4.

**Figure 6:**
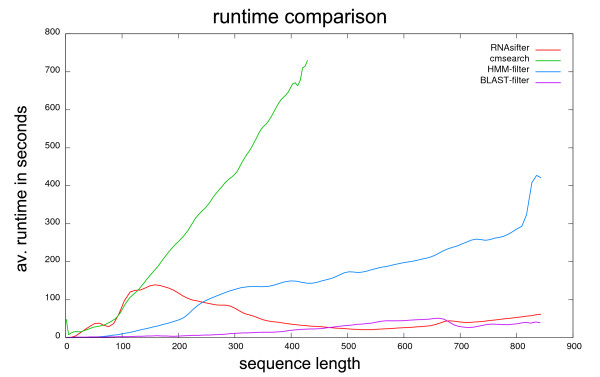
**Runtime comparison**. Average runtime in seconds vs. sequence length for the four different search methods: "cmsearch": for all *R *families in the database a cmsearch is executed until a match or all families were searched. "HMM-filter": The same as "cmsearch" but with activated "-hmmfilter" option. The measured time is just the runtime for the real search not for the construction of the HMM. We assume that this could be done once for a fixed Rfam release. "BLAST-filter": the Rfam Perl script "rfam_scan.pl" from the Rfam website is used. "RNAsifter" uses the k-best-shape-index with the suggested parameter set. The considered sequences for this comparison is the whole testset.

In the Filtering section we introduced two existing filtering approaches. Table 1 compares their asymptotical runtime with *RNAsifter *and the unfiltered procedure with *cmsearch*. Figure 6 illustrates the differences of measured runtimes for the four variants of retrieving the right families. Once more, the app. 10,000 sequences of the testing set are used for this analysis.

**Table 1:** Asymptotic runtimes. Asymptotic runtimes for the unfiltered *cmsearch *procedure and three filters, including our Sifter approach. *R *is the number of families in the current Rfam release. *L *is the target sequence length, *N *is the window size. |*f*| is the number of sequences in all families. *r*_*p *_is the remaining set of covariance models that have to be searched after the application of the filtering program *p*, *r *≤ *R*.

Program	Asymptotic runtime
CM	*O*(*R*·*L*·*N*^3^)
HMM	*O*(*R*·*L*·*N*^2 ^+ *r*_*HMM*_·*L*·*N*^3^)
BLAST	*O*(*R*·|*f*|·*N *+ *r*_*BLAST*_·*L*·*N*^3^)
Sifter	*O*(*N*^3 ^+ *r*_*Sifter*_·*L*·*N*^3^)

"BLAST-filter": the Rfam Perl script "rfam_scan.pl" from the Rfam website is used. "RNAsifter" uses the k-best-shape-index with the suggested parameter set. The considered sequences for this comparison is the whole testset.

## Conclusion

We have introduced the idea of shape-index based filtering for faster search in structural RNA databases. The approach is based on the use of family shape spectra, query shape spectra, and shape abstraction levels, each of which can be computed in different ways. Central to all combinations of these constituents is that the shape index can be accessed with exact matching techniques, which requires negligible computing time. Different parameter settings have been explored systematically, with a filtration ratio ranging from 0.025 to 0.28, and sensitivity ranging from 0.81 to 0.99. We recommend a particular setting with filtration ratio 0.15 and sensitivity 0.974. Not all possibilities have been explored yet. For example, one could work with family-specific parameters *k*_*family *_when constructing the index.

When (say) 15% of all family models must be searched, the practical speed-up depends on *which *models these are. miRNAs, for example, can be searched with CM models much faster than (say) RNaseP. Therefore, we cannot simply derive a 40-fold runtime speedup from a filtration ratio of 0.025. To provide a concrete example: The first 5003 ncRNA predictions from the RNAz screen [1] were matched against Rfam, and *RNAsifter *reduced runtime from 876.3 to 242.2 hours. Using the default parameters in this experiment, the filtration ratio is $\frac{1}{6}$, but runtime only decreases to $\frac{1}{4}$.

Two interesting observations can be drawn from the performance curve in Figure 1. Consider the relative position of the data points for (*k*_*family *_= 1, *k*_*query *_= 5, *ε *= 0.8) and (*k*_*family *_= 5, *k*_*query *_= 1, *ε *= 0.8), where the former is better both in terms of filtration ratio and of sensitivity. Hence, it is more important to consider multiple structures in the query than in the family. This has the plausible explanation that the several members in a family normally do not all fold into the same top-ranked shape, and this behaviour helps to find queries with similar behaviour. This means that structural variation within the family, to a certain extent, is positive information.

The other interesting observation suggested by the curve is that our method seems to hit the wall near 98% sensitivity. Assuming for a moment that shape-index based filtering was a perfect method, a keen conclusion would be that 2% of Rfam sequences are misclassified. However, we know that our filtering cannot be perfect when structures are classified using pseudoknot features, which currently cannot be handled by shape abstraction. A closer look at the missed cases shows that these are mainly due to the members of the "difficult" families. They include the families RF00177, RF00373, RF00009 (with and without pseudoknots); a full list of 25 "difficult" families in the present Rfam release is given in Additional file 1.

In a preliminary investigation of human ncRNA predictions from the screen by Washietl et al. [1], applied to 35985 hypothetical ncRNAs of high RNAz score, our filtering technique discovered 4 new Rfam hits. On the other hand, it overlooked 43 hits found in the original study. This can be traced back to cases where the candidate ncRNA was poorly embedded in the *RNAz *window. Naturally, our structure based approach is more sensitive to this than a filter that looks for a short stretch of sequence similarity. Working with an adaptive window size is a current research problem in RNA gene prediction. *RNAsifter *will benefit from advances in this direction.

Comparing the different filtering techniques, the method of choice depends on the scenario. If (say) a complete genome is to be scanned, the HMM-based filtering [9,10] provides a fast screening approach, where the faster HMMs must be run for each family, but only a few CM searches. Shape-index based filtering does not provide a screening mode, and can only be applied when individual ncRNA candidates have been predicted by (computational or experimental) methods. The two filters can also be used in cooperation: The shape index can be used to further restrict the number of CM searches that have to be performed as the result of positive HMM filtering. Conversely, the HMM family model could be run prior to a CM search triggered by a shape index hit. Here, we have presented the shape indexing technique in its pure form. The trade-offs achieved with filter combinations are a subject of future work.

second row family RF00017 is omitted, the third row omits families RF00017 *and *RF00230 and so on. Note that the testset shrinks, because the sequences of a skipped family are also removed from the set.

## Authors' contributions

RG had the initial idea for a shape filter. All three authors participated in the conceptual development of the approach. SJ analyzed the data and implemented all software. All authors read and approved the final manuscript.

## Supplementary Material

Additional file 1 **Effects of skipping "difficult" families on sensitivity and filtration ratio**. The test- and training sets are constructed as above, but this time we choose up to 1,000 sequences for each family instead of four. Random- and gene- testsets are not considered, because we focus on the changes of the sensitivity. *RNAsifter *is set to *k*_*family *_= 3, *k*_*query *_= 5, *ε *= 0.4. One has to read the rows in a accumulative fashion. In the first row no family is skipped, in the second row family RF00017 is omitted, the third row omits families RF00017 *and *RF00230 and so on. Note that the testset shrinks, because the sequences of a skipped family are also removed from the set. Click here for file
