# Supplementary material for: Shape based indexing for faster search of RNA family databases
Source: BMC Bioinformatics. 2008 Feb 29;9:131. doi: 10.1186/1471-2105-9-131 (PMC2277397; doi:10.1186/1471-2105-9-131)
Supplement: Additional file 1 — Effects of skipping "difficult" families on sensitivity and filtration ratio. The test- and training sets are constructed as above, but this time we choose up to 1,000 sequences for each family instead of four. Random- and gene- testsets are not considered, because we focus on the changes of the sensitivity. RNAsifter is set to kfamily = 3, kquery = 5, ε = 0.4. One has to read the rows in a accumulative fashion. In the first row no family is skipped, in the second row family RF00017 is omitted, the third row omits families RF00017 and RF00230 and so on. Note that the testset shrinks, because the sequences of a skipped family are also removed from the set. [file 1471-2105-9-131-S1.pdf]

skipped families	sensitivity	filtration ratio	# matches	# tested sequences	containing pseudoknots	common name
-	98.10%	0.79%	25,462	25,956		
RF00017	98.09%	0.80%	25,291	25,783	**yes**	SRP_euk_arch
RF00230	98.11%	0.79%	25,016	25,497	no	T-box
RF00210	98.17%	0.79%	24,956	25,422	no	IRES_Aptho
RF00028	98.73%	0.81%	24,112	24,422	**yes**	Intron_gpI
RF00134	98.73%	0.81%	24,096	24,405	no	snoZ196
RF00234	98.74%	0.81%	24,080	24,388	no	glmS
RF00228	98.77%	0.81%	24,034	24,334	no	IRES_HepA
RF00010	98.90%	0.81%	23,880	24,145	**yes**	RNaseP_bact_a
RF00018	98.92%	0.82%	23,868	24,128	no	CsrB
RF00513	98.93%	0.81%	23,857	24,116	no	Trp_leader
RF00100	98.95%	0.81%	23,795	24,048	no	7SK
RF00379	98.96%	0.81%	23,766	24,016	no	ydaO-yuaA
RF00040	98.96%	0.81%	23,757	24,006	no	rne5
RF00340	98.97%	0.81%	23,748	23,996	no	SNORA36
RF00216	98.98%	0.81%	23,730	23,975	**yes**	IRES_c-myc
RF00024	99.01%	0.81%	23,713	23,951	**yes**	Telomerase-vert
RF00023	99.12%	0.82%	23,601	23,811	**yes**	tmRNA
RF00135	99.13%	0.82%	23,589	23,797	no	snoZ223
RF00177	99.57%	0.82%	23,559	23,660	no	SSU_rRNA_5
RF00011	99.65%	0.82%	23,512	23,595	**yes**	RNaseP_bact_b
RF00009	99.70%	0.83%	23,481	23,551	**yes**	RNaseP_nuc
RF00373	99.76%	0.83%	23,457	23,513	**yes**	RNaseP_arch
RF00030	99.79%	0.83%	23,445	23,495	**yes**	RNase_MRP
RF00193	99.79%	0.83%	23,437	23,486	no	CTV_rep_sig
RF00552	99.80%	0.83%	23,429	23,477	no	rncO

Additional file 1: **Effects of skipping "'difficult"' families on sensitivity and filtration ratio.** The test- and trainingsets are constructed as above, but this time we choose up to 1,000 sequences for each family instead of four. Random- and gene- testsets are not considered, because we focus on the changes of the sensitivity. *RNAsifter* is set to $k_{family} = 4$, $k_{query} = 4$, $\epsilon = 0.3$. One has to read the rows in a accumulative fashion. In the first row no family is skipped, in the second row family RF00017 is omitted, the third row omits families RF00017 *and* RF00230 and so on. Note that the testset shrinks, because the sequences of a skipped family are also removed from the set. Two fifth of the listed families contain pseudoknots. We thinks that is the reason why they can hardly be searched, but we cannot explain the appearance of the others.
